# Curcumin-Loaded Drug Delivery Systems for Acute and Chronic Wound Management: A Review

**DOI:** 10.3390/bioengineering12080860

**Published:** 2025-08-11

**Authors:** Xiaoxuan Deng, Jithendra Ratnayake, Azam Ali

**Affiliations:** 1 Centre for Bioengineering & Nanomedicine (Dunedin), Faculty of Dentistry, Division of Sciences, University of Otago, P.O. Box 56, Dunedin 9054, New Zealand; christina.deng@otago.ac.nz; 2 Sir John Walsh Research Institute, Faculty of Dentistry, University of Otago, P.O. Box 56, Dunedin 9054, New Zealand; jithendra.ratnayake@otago.ac.nz

**Keywords:** wound healing, curcumin, wound management, hydrogels

## Abstract

Wound healing is a physiological process including haemostasis, inflammation, proliferation, and remodelling. Acute wounds typically follow a predictable healing process, whereas chronic wounds cause prolonged inflammation and infection, failing to progress through typical healing phases and presenting significant clinical challenges. A combination of wound care techniques and therapeutic agents is required to manage chronic wounds effectively. Curcumin is a bioactive compound derived from *Curcuma longa* and has gained attention for its potent antioxidant, anti-inflammatory, and antibacterial properties. The first part of this review aims to provide a comprehensive overview of the physiology of wound healing, focusing on the pathophysiology and management of acute and chronic wounds, followed by the biological activity of curcumin in wound healing, emphasising its impact on promoting tissue repair. Finally, this review explores curcumin-loaded dressings, such as hydrogels, nanofibrous membranes, polymeric micelles, and films, offering controlled drug release and targeted curcumin delivery to enhance wound healing.

## 1. Introduction

Wound healing is a complex and dynamic biological process, and several events, such as cellular, molecular, and biochemical, are involved in restoring the damaged tissue [1]. While acute wounds typically progress through these phases efficiently, chronic wounds often fail to heal due to a variety of factors, including persistent infection, excessive inflammation, and impaired cellular activity [2]. Chronic wounds, such as diabetic ulcers, venous leg ulcers, and pressure ulcers, pose a significant burden on healthcare systems worldwide due to prolonged treatment times, high recurrence rates, and the increased risk of complications [3]. Managing chronic wounds remains a clinical challenge, necessitating novel therapeutic approaches to promote efficient and complete healing.

Traditional wound care methods predominantly rely on synthetic and chemically derived agents, but there are existing drawbacks such as potential cytotoxicity, allergic reactions, and the growing problem of antimicrobial resistance. Therefore, there is a need for alternative solutions [4]. In recent years, there has been growing interest in the use of natural compounds as therapeutic agents for wound healing. Among them, curcumin, the primary bioactive component of *Curcuma longa* (turmeric), has demonstrated significant potential due to its multifaceted biological properties [5]. Curcumin exhibits strong antioxidant, antibacterial, and anti-inflammatory effects, all of which are crucial in mitigating the factors that impair wound healing. By reducing oxidative stress, controlling bacterial infections, and modulating the inflammatory response, curcumin helps create a favourable environment for tissue regeneration and repair [6].

Although curcumin possesses broad therapeutic potential [5], its clinical application remains limited due to several intrinsic physicochemical constraints. These include low aqueous solubility, poor gastrointestinal absorption, rapid metabolic transformation, and accelerated systemic elimination following oral administration—all contributing to its poor bioavailability [7,8]. Furthermore, curcumin is unstable under physiological conditions, undergoing rapid degradation, which further diminishes its clinical efficacy [9].

To overcome these limitations, various bioavailability-enhanced formulations have been developed. For instance, a clinical study comparing Curene^®^—a novel curcumin formulation—to a turmeric oil-based formulation (CP-01) and standard 95% curcuminoids demonstrated significantly improved oral bioavailability of Curene^®^, along with a favourable safety profile in healthy volunteers [10]. Similarly, Longvida^®^ Optimised Curcumin (LC) has been shown to exert anti-inflammatory effects in two-month-old GFAP-IL6 transgenic mice, reducing neuroinflammation and associated motor deficits [11].

Numerous commercial formulations with defined pharmacokinetic profiles have since emerged, including Meriva^®^, Longvida^®^, CurQfen™, MicroActive^®^, Micronized Curcumin, NovaSol^®^ (micellar curcumin), CurcuWin^®^, Biocurcumax™, and Curcumin C3 Complex^®^ with Bioperine, among others. Among these, NovaSol^®^ (185-fold), CurcuWin^®^ (136-fold), and Longvida^®^ (100-fold) have demonstrated exceptionally high bioavailability relative to standard curcumin [12]. Additional formulations such as CurcuminRich, Biomor, Liposomal Curcumin (including mango-flavoured variants), and Dr. Mercola Curcumin Advanced are also commercially available for oral administration [13].

Researchers have developed curcumin-loaded biomedical devices to address these limitations, enhance their stability, and achieve controlled delivery to the wound site. These devices include hydrogels, nanofibrous scaffolds, and films. They provide a platform for sustained curcumin delivery, ensuring that therapeutic concentrations are maintained at the wound site for extended periods [14].

This review aims to provide a comprehensive overview of the application of curcumin-loaded delivery systems in managing acute and chronic wounds, focusing on integrating curcumin bioactive molecules into advanced wound care systems through advanced fabrication techniques. By exploring the synergistic potential of natural compounds and cutting-edge technologies, this review highlights emerging trends and future directions in wound care, emphasising the role of curcumin-loaded medical devices as innovative and sustainable alternatives to conventional wound management strategies.

## 2. Physiology of Wound Healing

When the skin structure encounters physical disruption, it often results in fluid loss, injury, and pain. Wounds are categorised as acute or chronic, depending on the healing timeline and process [15]. Acute wounds, such as surgical incisions, abrasions, and minor cuts, typically heal within 8–12 weeks due to a well-coordinated cascade of growth factors, cytokines, and matrix proteins. In contrast, chronic wounds, like diabetic ulcers, require more than 12 weeks to heal and progress at a slower rate, failing to follow the normal wound healing process [16].

**Figure 1:**
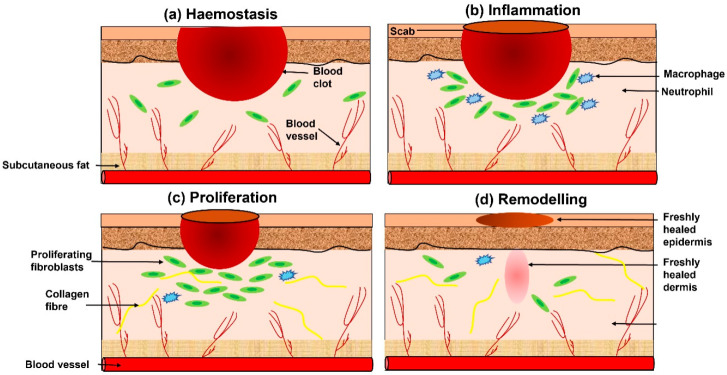
Schematic representation of wound healing process, including (**a**) haemostasis, (**b**) inflammation, (**c**) proliferation, (**d**) remodelling.

The healing of acute wounds involves the coordinated action of platelets, keratinocytes, immune cells, microvascular cells, and fibroblasts in a structured tissue repair process [1]. This process consists of four distinct phases: haemostasis/coagulation, inflammation, proliferation (granulation tissue formation), and remodelling (scar formation) (Figure 1).

Immediately after injury, platelet release to the damaged blood vessels initiates the haemostatic response (Figure 1a), creating blood clots to prevent excessive bleeding and protect the wound site [17]. Platelets then release key growth factors and cytokines, such as platelet-derived growth factor (PDGF) and transforming growth factors β1 and β2 (TGF-β1 and TGF-β2), which attract inflammatory cells like leukocytes, neutrophils, and macrophages to remove debris and eliminate foreign bodies and bacteria [18].

After the inflammatory phase, the proliferation phase begins (Figure 1c). During this phase, connective tissue and cells rapidly multiply, and the extracellular matrix (ECM)—including components like hyaluronan and proteoglycans—forms granulation tissue to replace the initial clot [19]. Various cytokines and growth factors (e.g., the TGF-β family, vascular epidermal growth factor, and interleukin family) are heavily involved in this phase, which can last several days to a few weeks before the healing process progresses to the final stage [16].

The remodelling phase (Figure 1d), or maturation phase, marks the point at which the wound achieves full closure. Plasmin plays a role in this phase by removing excess fibrin and recruiting keratinocytes to the wound site [20]. As collagen accumulates, a scar forms. Over time, collagen type III is gradually replaced by collagen type I until the normal skin ratio is restored [16]. Cross-linking of collagen fibrils continues to increase tensile strength in the wound area, and this phase can last several weeks to months [21].

## 3. Acute Wound Management

The three most infection-prone wound types are burns, surgical site wounds, and traumatic wounds [22]. Burn wounds are caused by heat, chemicals, electricity, or radiation [23]. Pathogens, including *Staphylococcus aureus* (*S. aureus*), *Escherichia coli* (*E. coli*), *Pseudomonas aeruginosa* (*P. aeruginosa*), and coagulase-negative *Staphylococci*, are common in the acute phase post-burn [24]. Surgical site wound infections (SSWIs) are caused by microorganisms introduced via sutures or prostheses [25]. These infections significantly increase morbidity and mortality. The primary pathogens from the epidermis and medical devices are *Staphylococcus epidermidis* and *S. aureus* [26]. Traumatic wounds, including abrasions and lacerations, involve extensive tissue, bone, or organ damage, which have *S. aureus* and *P. aeruginosa* as the predominant infectious agents [27]. Severe infections may lead to sepsis, necrotizing fasciitis, or diffuse septic peritonitis [28].

Traditional acute wound management includes wound dressing, antibiotics, and debridement. Wound dressings have been designed as temporary protective barriers to prevent or manage infections. However, they can potentially facilitate microbial colonization and biofilm formation, increasing the microbial load and delaying healing [29]. An ideal wound dressing should be flexible and provide a physical barrier while allowing oxygen exchange [30]. Antibiotics were discovered in the late 19th century, and agents such as sulfonamides, penicillin, streptomycin, tetracycline, and vancomycin have been widely used for infection control. However, the overuse of topical and systemic antibiotics, particularly in uninfected wounds or for prolonged periods, has contributed to a global antibiotic resistance crisis [31]. Debridement is the standard surgical intervention for wound infections, particularly in burn injuries. It reduces bacterial diversity, promotes epithelialization, and lowers microbial load by removing necrotic tissue, apoptotic cells, and biofilms [32]. In cases of deep infection involving muscle or adipose tissue, amputation may be necessary to prevent systemic infection or sepsis. High microbial loads can lead to complications such as osteomyelitis, ultimately necessitating amputation [33].

## 4. Chronic Wound Management

### 4.1. Chronic Wound Causes and Complications

Chronic wounds are commonly classified as vascular ulcers (including venous and arterial ulcers), diabetic ulcers, and pressure ulcers [34]. A key feature of chronic wounds is the prolonged or excessive inflammatory phase during healing, often accompanied by persistent infection, biofilm formation, and an inability to respond to reparative stimuli [35]. These pathophysiological conditions ultimately prevent the wound from healing. Furthermore, bacteria from the external environment, surrounding skin, or endogenous sources may enter and contaminate the wound. The immune response to bacterial presence can further prolong tissue inflammation, delaying the healing process. Invading pathogens can form biofilms, protecting bacteria from the immune system and increasing their resistance to antimicrobial agents, potentially leading to systemic infection [36].

**Figure 2:**
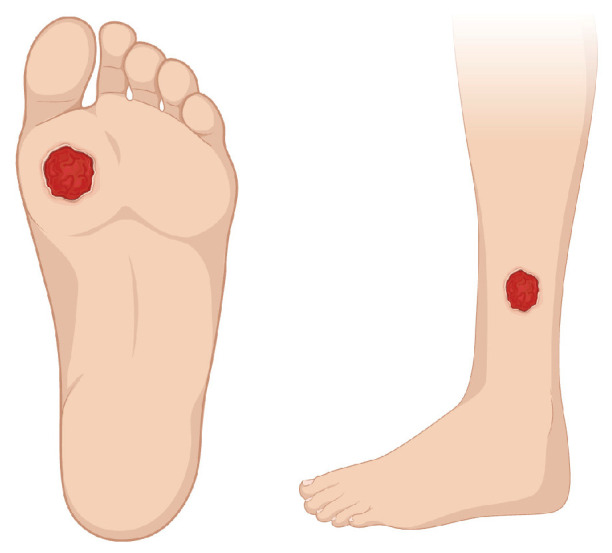
Diabetic ulcer in the foot.

One of the most significant conditions related to impaired wound healing is diabetes mellitus. Individuals with diabetes are highly prone to developing chronic wounds, particularly diabetic ulcers (Figure 2), which are the most prevalent type of chronic wound. These ulcers typically appear on the legs or feet and may necessitate hospitalisation or even amputation [37]. The relationship between diabetes and wound healing is complex, involving impaired oxygen delivery due to narrowed arteries, reduced intracellular bacterial killing (a key mechanism against pathogens), and diminished infection resistance caused by impaired neutrophil diapedesis and phagocytosis. Prolonged diabetes can reduce tissue oxygenation and damage blood vessels, resulting in chronic non-healing ulcers [38].

Venous ulcers, affecting mainly older patients, commonly target the lower limbs. These ulcers are caused by damage to the superficial and deep venous systems, leading to venous hypertension and reduced venous return [39]. Increased blood pressure makes vessel walls more permeable, causing leakage of fibre and other substances into perivascular spaces, eventually reducing oxygen supply to surrounding tissues. The diminished blood flow results in ischemia, negatively impacting wound healing in the affected region [38]. Additionally, the iron in haemoglobin is toxic and may lead to hemosiderosis and skin ulceration [40].

Pressure ulcers primarily affect elderly or paralysed individuals, particularly those suffering from spinal cord injuries. These ulcers often develop in areas under constant pressure, such as the heels, sacrum, and shoulder blades. Continuous pressure reduces oxygen diffusion to the underlying tissues, contributing to chronic ulcers [41].

Malnutrition is another factor associated with impaired wound healing. Inadequate nutrient intake prevents the body from maintaining healthy tissue and organ functions. Proper wound healing requires normal collagen synthesis and extracellular matrix formation, which depend on sufficient intake of proteins, vitamins, and minerals [42].

### 4.2. Pathophysiology of Chronic Wounds

A low level of reactive oxygen species (ROS) is generated during the normal wound healing process to combat pathogens and inhibit microbial infections. To prevent the toxic effects of ROS, enzyme systems in mammalian cells neutralise free radicals and repair oxidised molecules at the wound site. However, excessive ROS production disrupts the redox balance, leading to oxidative stress and prolonged inflammation, a key factor in the pathogenesis of chronic wounds [43]. For example, diabetic foot ulcers exhibit persistent inflammation, characterised by dysregulated and sustained infiltration of neutrophils and macrophages. These immune cells produce radical oxygen species to fight invading microbes, which can also provoke inflammation and cytotoxicity [44].

Abundant neutrophil infiltration at the wound site releases proteases, oxygen-free radicals, and inflammatory mediators such as TNF-α and IL-1 [20]. These factors cause DNA damage and inactivate enzymes like free-radical scavenging enzymes, further triggering the phosphorylation of phosphatidylinositol-3-kinase (PI3K)/Akt and activation of nuclear factor (NF)-κB. NF-κB then migrates to the nucleus, inducing the expression of various pro-inflammatory genes [45]. Elevated levels of inflammatory cytokines and collagenases, such as matrix metalloproteinases (MMP8), as well as gelatinases like MMP2 and MMP9, result in excessive tissue destruction, leading to non-healing wounds. In such cases, antioxidant therapy can help balance oxidant levels and restore redox homeostasis at the wound site [46]. Research has shown dysregulated TNF-α signalling and NF-κB overexpression in chronic wounds [47], highlighting the need for therapies that reduce hyper-inflammation and promote wound healing.

Chronic wounds are also highly susceptible to bacterial infections due to their prolonged open state, making them prone to colonisation by microorganisms [48]. Devitalised tissues can further promote pathogen proliferation. Both aerobic and anaerobic bacteria are commonly found contaminating chronic wounds. Aerobic pathogens such as *Staphylococcus aureus*, methicillin-resistant *Staphylococcus aureus* (MRSA), *Pseudomonas aeruginosa*, and *Streptococci* are frequently detected. At the same time, anaerobic bacteria like *Bacteroides*, *Prevotella*, *Porphyromonas*, and *Peptostreptococcus* are also common contaminants [49]. The low tissue oxygen levels in chronic wounds support the growth of anaerobic bacteria, and conventional clinical approaches often struggle to isolate these microbes, increasing the risk of further infection [50]. The development of bacterial biofilms within the wound bed significantly contributes to wound infections. Single or mixed species of bacteria form biofilms, and the extracellular matrix they produce helps shield them from the immune system and antimicrobial agents. Studies have shown that more than 60% of chronic wounds contain biofilms, compared to just 6% in acute wounds [50].

### 4.3. Management Techniques

Several factors, including the presence of necrotic tissue, underlying infections, ongoing trauma, neoplasia, high levels of matrix metalloproteinases, and poor blood supply, should be addressed to create an optimum environment for chronic wound healing. These elements are primary contributors to the failure of wounds to heal. Additional factors such as nutritional deficiencies, low temperatures, insufficient vitamin C, zinc deficiency, and hormonal imbalances also impede the healing process [51].

Treating infected chronic wounds requires wound debridement followed by antibiotic therapy. Surgical debridement, commonly done under anaesthesia, uses tools like a scalpel, curette, or hydrosurgery techniques. In contrast, mechanical debridement, which does not require anaesthesia, can be performed using ultrasound, gauze abrasion, or wet-to-dry dressings [52]. Enzymatic debridement involves chemically breaking down dead tissue with enzymes. Surgical debridement is often the initial approach, with enzymatic debridement used as needed afterwards.

After debridement, antibiotic therapy is necessary. Systemic antibiotics are typically prescribed for 10 days to treat invasive infections and hemolytic streptococci, while osteomyelitis or chronic wounds may require treatment lasting six weeks or longer [53]. Topical antibiotics, which are less cytotoxic and have minimal systemic absorption, can also be effective. However, only minocycline and gentamicin are Food and Drug Administration (FDA)-approved for topical application, and their use should be restricted to two weeks to reduce the risk of antibiotic resistance [52].

Managing diabetic foot ulcers involves controlling blood sugar levels, monitoring circulation, removing callous skin, and eliminating infections. In severe cases where tissue destruction cannot be controlled, amputation may be necessary. Treating venous leg ulcers requires debridement, antibiotic therapy, appropriate wound dressings, compression therapy, and calf-muscle exercises [54]. Pressure ulcers are managed by relieving pressure from the wound site, debridement, selecting proper dressings, and controlling infections [55].

Wound dressings perform several key functions, including protecting the wound, maintaining moisture, alleviating pain, and providing compression. They shield the wound from external damage and help prevent further injury. Maintaining the correct moisture balance is vital, as a dry wound bed can cause cell death, slow epithelial migration, and impede matrix formation [56]. Moist dressings also minimise hypertrophic scarring, reduce inflammatory cytokines, and help relieve pain [52]. For chronic wounds, moisture levels must be carefully regulated. Hydrocolloid and alginate dressings are effective in maintaining the proper moisture balance, while advanced dressings can also deliver antimicrobial agents like silver or iodine for enhanced protection [57].

## 5. Biological Activity of Curcumin in Wound Healing

### 5.1. Bioactive Curcumin

**Figure 3:**
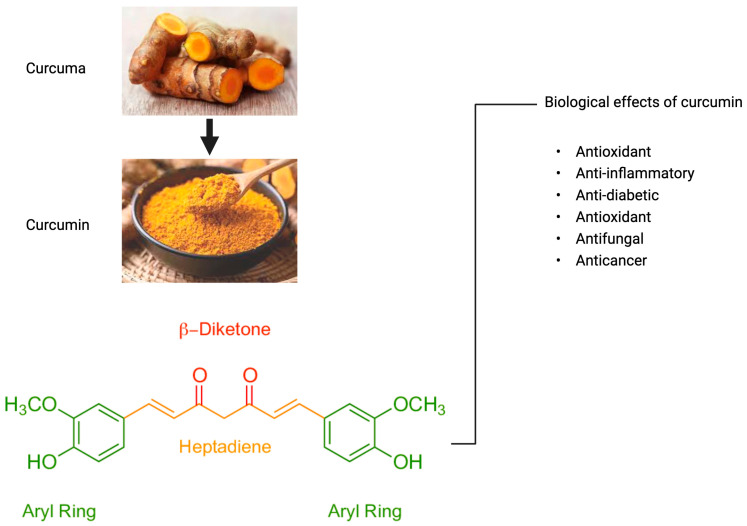
Chemical structure of curcumin and its biological effects.

The development of alternative wound healing products has recently become a popular topic. Natural sources have received increased attention. Nevertheless, there is still a lack of standardisation methods for the evaluation of the composition of natural products, which makes it difficult to estimate the efficacy of these compounds in wound management [58]. Curcumin is also known as diferuloylmethane, a natural-based polyphenol obtained from the rhizomes of *Curcuma longa*. The yellow pigments manifested by turmeric come from curcuminoids, and their biological activity is mainly due to the presence of curcumin. The curcumin structure was first proposed by Polish scientists in 1910. Two aromatic rings are connected by a seven-carbon linker, with methoxy and hydroxy groups at the ortho position, and an alpha, beta-unsaturated beta-diketone moiety (1,7-bis(4-hydroxy-3-methoxyphenyl)-1,6-heptadiene-3,5-dione) (Figure 3) [59]. The molecular formula of curcumin is C_21_H_20_O_6_. Curcumin exhibits keto-enol tautomerism due to the presence of the diketo group. Keto-enol tautomerism refers to the chemical equilibrium between a ketone or aldehyde (keto form) and an alcohol (enol) in organic chemistry. The keto form is more prevalent under slightly acidic and neutral conditions [60], while the enolic form is dominant under alkaline conditions [61]. In a solution, it displays cis–trans isomerism. The trans-form is more stable than the cis-form because of the difference in placement of two phenolic-methoxy groups on the backbone of curcumin.

Curcumin is a natural antioxidant that has been studied for its potential to aid in wound healing [58]. It can help accelerate wound healing by improving the rate of wound contraction. Studies have shown that it can enhance the wound healing rate by up to 20% [62]. Curcumin has been found to decrease inflammation and speed up wound healing, as demonstrated by Emiroglu et al. (2017) [63]. Additionally, Heydari et al. (2022) found that curcumin can promote the growth of new blood vessels and the deposition of collagen in chronic wounds [64]. Extensive animal studies have revealed that curcumin positively influences the formation of granulation tissue, collagen, and neovascularisation, thereby promoting the growth of fibroblasts [65,66,67]. Consequently, this leads to a noticeable increase in vascular density and the elimination of free radicals. Such exceptional attributes have made curcumin a highly effective treatment for chronic wounds, even among diabetic subjects [68].

Curcumin has attracted wide attention in the management of wounds due to its antimicrobial, anti-fungal, antioxidant, and anti-inflammatory activities. The U.S. Food and Drug Administration has labelled the curcumin molecule as “Generally Regarded as Safe” (GRAS). Despite its biocompatibility and non-toxicity, the biomedical application of curcumin is limited by its poor water solubility, and its absorption is limited in the gastrointestinal tract. To overcome this limitation, curcumin has been incorporated into polymer-based materials [16]. Nguyen et al. (2013) designed curcumin-loaded PLA nanofibers to function as wound-healing patches. The result showed that the addition of curcumin had a significant impact on the tensile stress of the nanofibers (which increased up to 3.5 MPa), which indicated its suitability as a wound dressing. In the in vivo study, dorsal wounds on rats achieved 87% and 99% wound closure rates on days 7 and 15, respectively [69]. Zahiri et al. (2020) produced a curcumin-loaded mat electrospun with PCL-gelatine. Sustained release of curcumin was observed from the electrospun mats, and the in vivo study revealed significant wound closure achievement of curcumin-loaded nanofiber mats on day 14 (82%) in comparison to the plain nanofibrous mats (73.4%) [70].

Studies have shown that curcumin facilitates wound healing by accelerating collagen and extracellular matrix synthesis [21,71]. Mahmoud et al. (2022) designed layered double hydroxides loaded with curcumin. They found that the collage production could begin within three days after applying curcumin-loaded material to a wound, and the process lasted 3 weeks, enhancing wound tension strength and resulting in faster wound healing [72]. Additionally, another study discovered that curcumin-loaded electrospun fibre could aggregate the wound exudate by pumping from the hydrophobic to hydrophilic fibre layers, which triggers a cascade release of curcumin that promotes collagen deposition and vascularisation [73]. Curcumin-sensitised ZnO nanoparticles were utilised for post-wound treatment. The product exhibited antibacterial and anti-inflammatory effects and accelerated wound contract, collagen deposition, and neovascularisation, as shown by Aslam et al. (2022) [74]. Moreover, treatment with curcumin + ZnO nanoconjugates led to increased collagen bundles within the dermis, angiogenesis, and remodelled collagen matrix [74]. Furthermore, curcumin can increase fibroblast distribution and shorten the inflammatory phase, accelerating re-epithelialization [75,76]. Ravanfar et al. (2022) observed that curcumin-PEG-loaded chitosan-gelatine nanoparticles significantly shortened the inflammatory phase and increased cellular proliferation in a burn wound rat model, and it also improved fibroblast distribution on the wound site and enhanced reepithelialisation [76].

### 5.2. Antioxidant Activity

Reactive oxygen species (ROS) play an important role in various cellular and metabolic activities [77]. They regulate intracellular signalling, apoptosis, cell differentiation, cell development, and immunity. However, ROS contain highly reactive chemicals such as superoxide radicals (O_2_−), nitrogen dioxide radicals (NO_2_−), hydroxyl radicals (−OH), and lipid peroxyl radicals (LOO−) [78]. These chemicals have the potential to cause inflammation and oxidative stress, which can impede the formation of granulation tissue and tissue remodelling—both critical in the wound healing process [79]. ROS also support the immune response in eliminating bacteria from wounds. Nonetheless, prolonged exposure to ROS can lead to oxidative stress, slowing down the wound-healing process and causing chronic wounds [79].

Reduction in ROS levels is demonstrated to be one of the mechanisms for curcumin’s therapeutic efficiency in wound healing [58]. Curcumin acts as a scavenger of free radicals and a modulator of lipoxygenases. Its unique chemical structure allows it to donate H-atoms and transport electrons, thereby exerting its antioxidant properties [80]. Curcumin also contains functional groups such as b-diketone and several p-electrons, contributing to its antioxidant capabilities by binding to metals and scavenging ROS. Curcumin also demonstrates radical scavenging abilities against various free radicals, including DPPH (2,2-diphenyl-1-picrylhydrazyl), ABTS (2,2′-azino-bis(3-ethylbenzothiazoline-6-sulfonic acid)), and DMPD (N,N-dimethyl-ρ-phenylenediamine) [81].

Extensive research underscores the antioxidant capabilities of dietary curcumin, highlighting its ability to scavenge hydrogen peroxide, chelate ferrous ions, and reduce ferric ions [82]. At a concentration of 15 μg/mL, curcumin inhibited 97.3% of lipid peroxidation, significantly more effective than α-tocopherol, a well-known antioxidant, which inhibited only 84.6% at a concentration of 45 μg/mL [83]. Furthermore, the phenolic OH group in curcumin is crucial for its antioxidant activity, capable of donating a hydrogen atom to neutralise free radicals [84,85]. In animal models, oral and topical curcumin administration increased the activity of antioxidant enzymes, such as superoxide dismutase, catalase, and glutathione peroxidase, which are critical for reducing oxidative stress and maintaining cellular health [86,87].

Moreover, curcumin enhances the production and function of antioxidants like glutathione (GSH) through the activation of the antioxidant Nrf2 pathway, an important cell-protective signalling pathway in wound healing [88]. Curcumin activates this pathway by modifying the reactive cysteine residues of Keap1, which reduces E3 ligase activity and allows Nrf2 to accumulate in cells [89].

The potential for incorporating curcumin into biomaterials for wound healing was highlighted in a study by Liu et al. (2018) that evaluated the effects of a curcumin-loaded gelatine hydrogel on skin healing [90]. In vitro testing revealed that curcumin can expedite wound healing by mitigating oxidative stress and injury to the wound site through its antioxidant properties. Curcumin stimulated angiogenesis, leading to the formation of new blood vessels and facilitating the transport of vital nutrients and oxygen to the wound site. Additionally, curcumin fostered cell proliferation, generating new cells and boosting collagen production, the chief protein component of the skin, at the wound site. The in vivo findings on diabetic mice demonstrated that using the curcumin hydrogel was more effective than the control hydrogel. The curcumin hydrogel notably reduced wound size, inducing new blood vessel formation and augmented collagen formation at the wound site [90]. These results suggest that curcumin-loaded biomaterials have the potential to enhance wound healing and promote tissue regeneration.

### 5.3. Anti-Inflammatory Activity

Inflammation is a complex response in the body that is necessary for wound healing. It is the natural way for the body to respond to an injury or infection, and it plays a fundamental role in the early stages of tissue repair. However, excessive inflammation can cause tissue damage and chronic diseases, which makes it crucial to control inflammation.

Curcumin possesses potent anti-inflammatory properties. One of the mechanisms by which curcumin exerts its anti-inflammatory effects is by reducing the production of several inflammatory cytokines, such as interleukin (IL)-1, IL-6, IL-8, and tumour necrosis factor-alpha (TNF-α), which are small proteins that play a vital role in the inflammatory response. Specifically, curcumin reduces the production of two critical cytokines, TNF-α and IL-1, which are generated by monocytes and macrophages and are known to trigger inflammation [91].

Curcumin inhibits the activity of NF-(κ)B, a transcription factor that plays a critical role in initiating inflammatory responses. Normally, NF-(κ)B is activated by a variety of kinases, including AKT, PI3K, and IKK. By inhibiting NF-(κ)B, curcumin may help reduce the inflammatory processes in the body [92]. Besides its effects on NF-(κ)B, curcumin also acts through other signalling pathways to combat inflammation. For example, curcumin can enhance PPAR-γ activity by hindering vascular smooth muscle cell proliferation to decrease angiotensin II-induced inflammation [93,94]. In addition, it has also been demonstrated to impede the TLR4-MD2 signalling complex by taking over LPS for MD2 binding to reduce inflammation [95].

Alibolandi and their team developed dextran hydrogels that contained curcumin nano-micelles [96]. The hydrogels absorbed water over 8 days before degrading due to cross-linkage hydrolysis. When tested on mice, the wounds treated with curcumin-loaded hydrogels showed significant reduction after 21 days of treatment, likely through the sustained release of curcumin through the wound dressing to decrease inflammatory responses [96]. In another study, Zhao and co-workers developed innovative hydrogels to treat infections in cutaneous wounds [97]. The in vivo animal studies showed that the curcumin-loaded hydrogel had a faster healing rate on day 14 compared to plain hydrogels and gauze-treated wounds. The hydrogel also inhibited the NF-(κ)B signalling pathway and reduced oxidative stress, leading to a reduction in inflammatory responses to microbial infections [97]. Similarly, Merrell et al. (2009) developed PCL nanofibers loaded with curcumin [92]. A sustained curcumin release from the nanofibers for three days under physiological conditions showed that the quantity of curcumin released remained below the known cytotoxic concentration, ensuring its therapeutic potential. Additionally, the prepared nanofibers demonstrated a reduced inflammatory response against rat monocyte macrophages in inflammatory studies, suggesting their potential to reduce inflammation [92]. In addition, Mohammadi et al. (2019) formulated nanofibers composed of PCL-PEG and imbued with chrysin-curcumin to accelerate the wound healing process [98]. In vivo experiments conducted on male rats unveiled an upsurge in the expression of the IL-6 gene, which plays a crucial role in inflammation, on day 10. Additionally, the study evidenced a marked reduction in the expression of matrix metalloproteinase 2 (MMP-2) and downregulation of iNOS, which are vital in the healing process [98].

### 5.4. Antibacterial Activity

Curcumin is a natural compound with antibacterial properties. It prevents bacteria from attaching to host receptors by disrupting the bacterial quorum-sensing regulation system. Additionally, curcumin damages cell membranes, DNA proteins, and other cell structures of bacteria, which inhibits bacterial growth [99]. Researchers have employed various techniques to incorporate curcumin into wound dressings and other materials to create effective antibacterial agents for medical use. Studies suggest that curcumin has a negative effect on both Gram-negative and Gram-positive bacterial cells by damaging the permeability of the cytoplasmic membrane and the ATP-binding cassette transporters [100].

For instance, Ranjbar-Mohammadi et al. (2016) developed wound dressings using electrospun curcumin-loaded PCL and gum tragacanth. These dressings, with a curcumin content of 3 wt%, demonstrated antibacterial properties of 85.14% and 99% against extended-spectrum beta-lactamase (ESBL) and methicillin-resistant Staphylococcus aureus (MRSA), respectively, and they showed promising results in healing full-thickness wounds in rats [101]. Similarly, Sharma et al. (2022) constructed curcumin-loaded PVA and dip-coated PLA/PCL blend-based electrospun mats. These mats exhibited significant antibacterial activity against *E. coli* and *S. aureus*, with an adherent bacteria rate of less than 10% [102].

The curcumin-incorporated hybrid constructs exhibited the ability to target bacterial cells and disrupt their membrane integrity. For instance, Dos Santos et al. (2021) employed a combination of coaxial electrospinning and 3D printing techniques to design core-sheath electrospun nanofibers containing PEO/curcumin/tetracycline hydrochloride and zein/PCL/beta-glycerolphosphate. The product demonstrated excellent antibacterial activity [103]. Chagas et al. (2021) designed three layered nanofibers based on curcumin-loaded PLA and natural rubber, which prevented curcumin from photodegradation and provided protection against bacterial infiltration for 10 days [104]. Jose et al. (2022) fabricated 3D porous aerogels for chronic wound healing using curcumin-loaded cellulose nanofibers. The aerogels demonstrated excellent antimicrobial activity against pathogenic microorganisms [105]. Zhang et al. (2023) utilised a coating method to produce antibacterial surgical sutures decorated with curcumin-loaded zeolitic imidazolate framework-8. These sutures demonstrated significant antibacterial activity against *E. coli* and *S. aureus* [106]. Cai et al. (2023) encapsulated curcumin into ammonium alginate/PLA hydrogel, demonstrating an antibacterial rate exceeding 90% against *E. coli* and *S. aureus* [107]. Tyagi et al. (2015) studied the antibacterial activity of curcumin against *Staphylococcus aureus*, *Enterococcus faecalis*, *Escherichia coli*, and *Pseudomonas aeruginosa*. They found that curcumin caused membrane leakage in Gram-negative and Gram-positive bacteria, leading to cell death [108]. Finally, Zhang et al. (2021) fabricated curcumin/celecoxib electrospun membranes to prevent peri-tendinous adhesion. These membranes exhibited good antibacterial properties, with the effectiveness of the product primarily dependent upon the concentration of curcumin to bacteria [109].

### 5.5. Safety of Curcumin and Clinical Trials

Curcumin has been widely studied and is generally regarded as safe for both systemic and local use, with tolerability demonstrated even at high doses of up to 12 g/day [110,111]. It is recognised as “Generally Recognised as Safe” (GRAS) by the U.S. Food and Drug Administration [5]. Nonetheless, adverse effects, though infrequent, have been reported. Co-administration of curcumin (8000 mg/day) with gemcitabine (1000 mg/m^2^ weekly, IV) has led to gastrointestinal discomfort in some patients [112], which may be alleviated through concurrent antacid use [113].

Additionally, curcumin supplementation has been associated with iron deficiency anaemia, warranting monitoring in long-term users [114]. Although curcumin is often cited for its hepatoprotective effects [115], a systematic review of Italian case reports revealed instances of hepatotoxicity at daily doses ranging from 250 to 1812.5 mg over durations of 2 weeks to 8 months [116]. The precise mechanism behind curcumin-induced liver injury remains unidentified.

The most commonly reported side effects of curcumin include mild gastrointestinal symptoms—such as diarrhoea, abdominal pain, dyspepsia, nausea, flatulence, constipation, vomiting, and yellow stools—alongside occasional headache and dizziness. Rare but serious adverse events have also been noted, including cachexia and muscle wasting in a pancreatic cancer patient [117], and acute kidney injury following perioperative administration during abdominal aortic aneurysm repair [118]. Other uncommon events include hair loss, low-grade fever, and throat infection.

Given curcumin’s poor oral bioavailability, approximately 55% of turmeric supplements in the U.S. market incorporate bioavailability-enhancing strategies, including lipid-based carriers or piperine co-formulation to inhibit metabolism [119]. Reflecting this trend, 45% of curcumin clinical trials have evaluated such enhanced formulations. Notably, these were most frequently studied in musculoskeletal conditions, with fewer applications in gastrointestinal disorders, likely due to the local (rather than systemic) targeting in most gastro-intenstinal studies. However, variability in bioavailability-enhancing technologies, coupled with limited pharmacokinetic evaluation, complicates cross-study comparisons of curcuminoid dosing. Consequently, dose standardisation across clinical trials remains challenging, and dose data were not uniformly analysed. A randomised controlled trial conducted by Mokhtari et al. to determine the effects of curcumin intake on wound healing in patients with diabetic foot ulcer showed that administering nanocurcumin did not affect the wound healing parameters [68]. In another study, when curcumin, apple honey, and placebo creams were applied to the episiotomy wound, both apple honey and curcumin promoted episiotomy wound healing compared to the placebo. However, the result was not statistically significant [120].

## 6. Curcumin-Loaded Delivery Systems for Wound Healing

Wound healing is a complex biological process that requires attentive management, such as cleaning the wound site, inhibiting microbial infections, preventing foreign substances, and selecting suitable wound dressings. A desirable wound dressing should meet several requirements, including high porosity, elimination of excess extrudates, limited bacterial growth, the transmission of gases, mechanical protection, and the ability to maintain a moist environment but not macerated [16]. An optimal environment is essential for rapid wound healing. Wound dressings can provide protection and moisture for wound beds and inhibit bacteria growth and infection.

The successful development of wound dressings involves the combination of biopolymers and synthetic polymers, as biopolymers alone suffer from poor mechanical properties, which can be modulated by the addition of synthetic polymers. The most commonly used biopolymers include hyaluronic acid, alginate, cellulose, chitosan, fibrin, collagen, gelatine, etc. They are biocompatible, biodegradable, bioadhesive, non-toxic, and have excellent haemostatic effects [121,122,123]. The synthetic polymers commonly used in wound dressing development include poly(ethylene glycol) (PEG), poly(vinyl pyrrolidone) (PVP), poly(vinyl alcohol) (PVA), poly(lactic-co-glycolic acid) (PLGA), polyglycolic acid (PGA), polylactide (PLA), poly(ε-caprolactone) (PCL), etc. Synthetic polymers exhibit excellent mechanical properties but generally have poor biocompatibility when combined with biopolymers, resulting in improved physicochemical properties [16].

**Figure 4:**
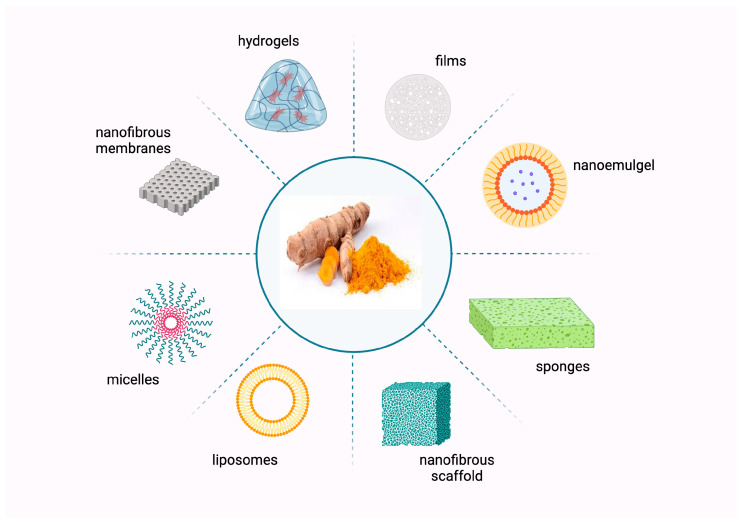
Curcumin-loaded wound dressing formulations.

Recently, there has been a surge of interest in the development of advanced materials for skin wound healing. This includes creating biocompatible films, polymeric micelles, hydrogels, and nanofibrous scaffolds containing curcumin (Figure 4). These biomedical devices have demonstrated the potential to facilitate wound healing and have been used to treat acute and chronic wounds [124,125].

### 6.1. Nanofibrous Scaffolds

Nanofibrous scaffolds are artificial extracellular matrices that can mimic the natural environment. They have gained more attention from researchers in recent years due to the large surface area-to-volume ratio and their versatile ability to load various bioactive compounds, which can provide a biomimetic environment for cell proliferation and migration and facilitate the wound healing process. Nanofibrous scaffolds can be fabricated with natural or synthetic polymers via phase separation, melt blowing, self-assembly, and electrospinning [126,127,128]. Amongst them, the electrospinning technique has been widely used to produce nanofibrous scaffolds for wound healing purposes because the electrospun products can prevent moisture loss, inhibit microbial growth, and remove exudates from the wound site. The unique properties of nanofibrous scaffolds make them potential candidates for treating acute and chronic wounds [50,129].

A curcumin-loaded nanofibrous scaffold is created through sequential electrospinning. This nanofibrous membrane contains three layers, each serving a distinct purpose. The bottom layer of the dressing is made up of a combination of gelatine, chitosan, and PCL. These components are chosen for their ability to effectively promote haemostasis and absorb exudate from the wound. Additionally, they help in creating an optimal moist environment for the wound, which is conducive to the healing process. The middle layer of the material is designed to release curcumin. This helps to effectively reduce oxidative stress and inflammation in the wound area. The top layer consists of silver nanoparticles, silk fibroin, and PCL. The silver nanoparticles are released from this layer to actively combat and eliminate external bacteria, contributing to the overall wound-healing process [130]. In another study, an eggshell membrane, a delicate protein layer situated between the egg white and the shell, was incorporated into the bottom layer of PVA alongside curcumin nanoparticles. The eggshell membrane exhibited significant antioxidant and anti-inflammatory properties, effectively facilitating the migration of dermal fibroblasts [131] (Table 1). This innovative approach to wound healing demonstrated promising results by enhancing exudate absorption, releasing therapeutic components, and promoting the deposition of extracellular matrix, thus contributing to the overall healing process [131,132].

### 6.2. Hydrogel

Hydrogels are hydrophilic polymeric gels that can swell in water and carry huge amounts of water while retaining the 3D structure [133]. Hydrogel dressings have a relatively higher swelling capacity and better biocompatibility in comparison to conventional wound dressings [134] and can provide hydrophilicity to the wound bed to maintain a moist environment and improve wound healing [135]. Additionally, hydrogel dressings and phytochemicals can be utilised as drug delivery systems. The application of silver–curcumin hydrogel dressings has been shown to inhibit *Escherichia coli* (*E. coli*) [136] (Table 1).

Topical application of curcumin-loaded hydrogels has been identified as an effective approach for treating chronic wounds. Li et al. loaded curcumin in a nanocarrier and incorporated it into the N,O-carboxymethyl chitosan/oxidised alginate hydrogel [137]. The hydrogel was subsequently applied in the full-thickness animal model, and the results showed significantly accelerated wound healing with complete wound closure within 14 days [138]. In another study, Gong et al. incorporated curcumin-loaded micelles into a hydrogel system, which showed strong tissue adhesion and prolonged curcumin release [139]. An in vivo animal study with the application of the hydrogel demonstrated accelerated wound healing by stimulating angiogenesis, improving collagen organisation, and promoting granulation tissue formation (Table 1) [139].

A recent study developed a hydrogel by loading chitosan, Pluronic 123, and curcumin to create a single-loaded formulation. The incorporation of gelatine resulted in a dual-loaded hydrogel. The findings indicated that the dual-loaded hydrogel exhibited enhanced water absorption capacity. Additionally, the curcumin release profile demonstrated a more sustained release from the dual-loaded hydrogel, attributed to the denser structure introduced by gelatine. In vivo studies further revealed that the application of the curcumin-loaded hydrogel significantly accelerated wound healing compared to untreated wounds [140].

### 6.3. Films

Film dressings, commonly used to promote wound healing, provide a moist environment conducive to tissue repair. Chen et al. developed curcumin-encapsulated chitosan–polyethylene glycol–silver nanoparticles (Chitosan-PEG-AgNPs) films, achieving an encapsulation efficiency of 69.9 ± 0.3% for curcumin. The swelling capacity of the curcumin-loaded film was 35.3% in 0.15% phosphate-buffered saline. The films exhibited significant antibacterial activity against *Staphylococcus aureus* and *Escherichia coli*, with notable inhibition observed at 24 and 48 h (Table 1). Furthermore, in vivo studies using a rat wound model demonstrated enhanced wound healing in the curcumin-loaded film group. The results revealed that these films accelerated the rate of epithelialisation and completed the process more rapidly compared to both control and non-curcumin-loaded films [141].

In a recent study, Chiaoprakobkij et al. developed a curcumin-loaded cellulose-alginate-gelatin film. The study demonstrated that the fluid uptake capacity of the films decreased as the curcumin loading increased. Similarly, higher curcumin content negatively impacted the films’ mechanical properties. While no significant curcumin release was observed in phosphate-buffered saline or artificial saliva, the curcumin-loaded films exhibited cytotoxic effects against oral cancer cells while showing no cytotoxicity toward HaCaT and gingival fibroblast cells. Additionally, the curcumin-loaded films displayed considerable antibacterial activity against both *Escherichia coli* and *Staphylococcus aureus*, with a more pronounced inhibitory effect against the Gram-positive *S. aureus* compared to the Gram-negative *E. coli* (Table 1) [142].

### 6.4. Polymeric Micelles

Polymeric micelles are self-assembling colloidal structures formed by amphiphilic block copolymers, featuring a core/shell architecture and typically measuring between 10 and 100 nm in size. Their adaptable and inherent characteristics, including biocompatibility, durability, excellent stability in both in vitro and in vivo environments, and ability to target pathological areas with impaired vasculature, make them highly suitable for drug delivery [143]. These micelles can effectively encapsulate poorly soluble drugs, such as curcumin, thereby enhancing their wound healing potential for treating cutaneous, diabetic, and excisional wounds (Table 1) [139].

The incorporation of micelles into hydrogel systems enables the formation of a dual microenvironment comprising both hydrophilic and hydrophobic domains. The hydrophobic cavities in the micelles enable micelle-containing hydrogels to encapsulate poorly water-soluble compounds and facilitate controlled drug release [144].

Zhang et al. created nano-micelles using curcumin and alginate to improve the bioavailability of curcumin. The results indicated that the micelles had an average size of 55.5 nm. Toxicology findings also demonstrated that loading curcumin at 7.5 mg had no adverse effects on cell viability. Additionally, at minimal curcumin concentrations of 53, 245, and 319 (g/mL), the micelles were effective against *Staphylococcus aureus*, *Pseudomonas aeruginosa*, and *Escherichia coli*. Furthermore, the micelles showed increased protein and collagen at the wound site and increased TGFβ1 expression, suggesting that the curcumin-loaded micelles can expedite wound healing [145]. In another study, Akbar et al. utilized the thin-film hydration method to fabricate curcumin-loaded micelles using chitosan, alginate, maltodextrin, pluronic F127, pluronic P123, and Tween^®^80. The results showed that these micelles reduced blood glucose levels and improved the lipid profile (Table 1). Additionally, the rats’ body weight remained stable after surgery, and various biochemical parameters indicated an accelerated wound healing process, indicating that these curcumin-loaded micelles have therapeutic potential and healing efficacy [146].

**Table 1:** Summary of different topical formulations of curcumin for wound healing.

Dressing Type	Composition	Preparation Method	Study Method	Key Findings	References
Nanofibrous membrane	Curcumin, honey, PVA, cellulose acetate	Electrospinning	In vitro	Curcumin and honey were encapsulated in PVA and cellulose acetate individually and also together. It was found that the dressings facilitated cellular activities and provided antimicrobial activity against common infection.	[147]
	Curcumin, gelatine, sodium bicarbonate, honey	Electrospinning	In vitro and in vivo with Wistar male albino rat model	The results of antioxidant and antibacterial activities showed better outcomes with the addition of curcumin and honey. In vivo study showed healed wounds on day 17.	[148]
	Curcumin, chitosan, gelatine, PCL	Electrospinning	In vitro and in vivo with rat dorsal skin defects model	In vitro studies demonstrated that the product exhibits strong antioxidant and antibacterial activity. In vivo studies showed it promotes granulation tissue formation, collagen deposition, and remodelling of epithelial tissue. Additionally, it accelerates wound healing by enhancing the expression of CD31 and TGF-β in the early stages of the healing process.	[130]
	Curcumin, surfactin, PCL, gelatine	Electrospinning	In vitro and in vivo with male Wistar rat model	In vitro studies demonstrated that the dressing exhibited over 99% antibacterial activity after 24 h. An increase in curcumin concentration resulted in reduced elastic modulus and increased tensile strength. In vivo studies demonstrated a significant improvement in the healing rate compared to control groups lacking curcumin.	[149]
	Curcumin, heparin, PLGA	Electrospinning	In vitro and in vivo with diabetic Sprague Dawley rat model	The dressings possessed high tensile strength and low cytotoxicity, along with increased hydrophilicity. In in vivo studies, the dressings were found to accelerate the re-epithelialization rate, promote higher angiogenesis, and enhance collagen deposition at the wound site.	[150]
	Curcumin, AgNPs, chitosan, polyethylene oxide	Electrospinning	In vitro and in vivo with male Kunming mouse model	This product demonstrated effective activity against both Gram-negative and Gram-positive bacteria in in vitro studies. In vivo studies showed improved wound closure rates compared to the commercial product AquacelAg.	[151]
Nanofibrous scaffold	Curcumin, carboxymethyl guar gum, graphene oxide	Electrospinning	In vitro and in vivo with rabbit model	In vitro wound healing assays using 3T3 L1 cell lines demonstrated 100% wound closure within 48 h. In vivo studies revealed that the nanofibrous scaffold containing curcumin exhibited antibacterial, anti-inflammatory, and antioxidant effects on chronic wounds.	[152]
	Curcumin, cellulose acetate, poly (ε-caprolactone)	Electrospinning	In vitro	Curcumin has a dual role as a drug and as a hydrophilicity-enhancing agent because of the formation of hydrogen bonds between its components. This enhances the swelling capacity by around 700% or 950%, depending on the percentage of added curcumin. The medicated scaffolds that were created increased the expression of actin in fibroblasts compared to the unmedicated ones.	[153]
	Sodium alginate and collagen	Physical mixing	In vitro and in vivo with female rat model	In vivo studies showed that the scaffold loaded with curcumin had a 90% wound healing rate at day 14 compared to 80% when a scaffold without curcumin was applied.	[71]
Nanofibrous mat	Curcumin, PCL, PVA, silk fibroin	Electrospinning	In vitro and in vivo with streptozotocin-induced diabetic mice model	Diameters of fibres: 200–350 nm. Tensile strength: 12.41–16.80 MPa. The product demonstrated faster wound healing compared to traditional formulations and has significant potential for healing diabetic wounds.	[154]
Nanoemulgel	Curcumin, Labrafac PG, Tween^®^ 80, PEG 400	Ultrasonic emulsification method	In vitro and in vivo with Wistar rat model	Droplet size: 56.25 ± 0.69 nm. Polydispersity index: 0.05 ± 0.01. Zeta potential: −20.26 ± 0.65 mV. The selected nanoemulsion was integrated into a 0.5% Carbopol^®^ 940 hydrogel matrix to create nanoemulgels for topical use. The developed curcumin nanoemulgel displayed thixotropic rheological behaviour and demonstrated a significant improvement in skin penetrability compared to curcumin dispersed in a traditional hydrogel system. The nanoemulgel design exhibited outstanding skin penetrability and showed promising wound healing capabilities in in vivo animal studies.	[155]
	Curcumin, resveratrol	Emulsification	In vitro and in vivo with burn-induced male Wister rat model	Particle size: 167–180 nm. Zeta potential: −17 to −20 mV. In vivo studies have shown the enhanced burn healing potential of the combination of nutraceuticals, as well as the promising delivery characteristics of the nanoemulgel dosage form.	[156]
Nanoemulsion	Curcumin, clove oil, Tween^®^ 80, PEG400	Ultrasonic emulsification method	In vitro and in vivo with Albino rat model	The optimised curcumin-loaded nanoemulsion was non-toxic and had a drug content of 98.11 ± 0.16%, pH of 7.4 ± 0.07, zeta potential of −11.67 ± 0.11, refractive index of 1.71 ± 0.034, and viscosity of 37 ± 7 cp. In addition, this nanoemulsion improved wound healing in rats by promoting the proliferation of epithelial cells and demonstrated significant anti-inflammatory effects in a rat model.	[157]
Nanocomposite	Curcumin, zinc, aluminium	Chemical precipitation	In vitro and in vivo with male albino rat model	In vitro drug release: 56.78 ± 1.51% within 24 h. In vivo studies demonstrated excellent wound healing capabilities, high muscle tensile strength, and strong anti-inflammatory properties.	[72]
Film	Curcumin, chitosan, PEG, AgNPs	Chemical ross-linking	In vitro and in vivo with Wistar albino rat model	Particle size: 13.48 nm. The viability of Vero cells reached 96.5% with a curcumin concentration of 100 μg/mL. The dressing exhibited improved inhibition of *S. aureus* and *E. coli* at 24 h and 48 h. Additionally, a wound contraction of 98% was observed on day 12.	[141]
	Curcumin, bacterial cellulose, alginate, gelatine	Mechanical blending and casting	In vitro	Water contact angles: 50–70°. Water vapour permeability: 300–800 g/m^2^/24 h. The curcumin-loaded films were non-toxic to human keratinocytes and had antibacterial activity against *E. coli* and *S. aureus*, with enhanced fluid uptake capability up to 700%.	[142]
Hydrogel membrane	Curcumin, chitosan, sodium alginate	Physical cross-linking	In vitro and in vivo with male Sprague Dawley rat model	In vitro drug release: 41 ± 4.2% within 24 h. Tensile strength: 16 MPa. In vivo testing showed 75 ± 2.3% reepithelialisation within 14 days compared to gauze-covered wounds.	[158]
Thermosensitive hydrogels	Curcumin, poloxamer 188, poloxamer 407	Cold swelling	In vitro and in vivo with Sprague Dawley rat model	Pore size: 5 to 10 μm. Live/dead assay showed that the curcumin-containing hydrogel extracts were non-toxic to cells. In vivo studies demonstrated the hydrogel’s ability to promote wound healing.	[159]
Hydrogel	Curcumin, chitosan, Lipoid S 100, polysorbate 20, stearylamine, sodium deoxycholate	Film hydration, hand-stirring	In vitro and ex vivo with full-thickness human skin model	Liposomes with a positive charge, created using stearylamine as a positive charge promoter, exhibited superior bioadhesion and improved, sustained penetration through full-thickness human skin compared to neutral and anionic liposomes.	[160]
Sponge	Curcumin, cellulose, β-cyclodextrin, chitosan	Synthesis, cross-linking, freeze-drying	In vitro	Binding with β-cyclodextrin increased the solubility of curcumin. Adding cyclodextrin complex and chitosan improved the sponge’s mechanical properties. The sponge was non-toxic to NCTC L929 and NHDF cells and showed increased antibacterial activities with the addition of chitosan. The authors believed that it could be used for chronic wounds.	[161]
Liposomes	Curcumin, Pluronic F127, liposomes	Encapsulation	In vitro	The impact of curcumin-loaded liposomes on a human keratinocyte cell line was examined, revealing no effect on cell viability. However, curcumin-loaded liposomes were found to enhance the cell migration rate and increase the expression of nuclear factor erythroid-related factor 2 and kelch-like erythroid cell-derived protein 1. This indicates a promising formulation for improved wound healing.	[162]
Nanoparticles	Curcumin, oleic acid, silica gel 60, Carbopol-934	Sonication, physical mixing	In vitro and in vivo with male Wistar rat model	Mesoporous silica loaded with curcumin was created by simply mixing a curcumin solution with mesoporous silica powder at 50 °C. In vivo rat studies had two groups. One group was treated with curcumin-loaded mesoporous silica, while the other group was treated with sulfadiazine. The curcumin-treated group displayed more significant improvements in the healing process, attributed to the formulation’s anti-inflammatory effects and its capacity to enhance angiogenesis, epithelization, and collagen synthesis.	[163]
Carbon dots	Curcumin, carbon dots, ethylenediamine, bovine gelatine (Type B)	Cross-linking	In vitro and in vivo with male Sprague Dawley rat model	The solubility and stability of free curcumin were enhanced by formulating it as carbon dots. Carbon dots exhibited improved proliferative, proangiogenic, and antibacterial activity, making them suitable for wound healing applications. In vivo studies demonstrated accelerated wound contraction, increased angiogenesis, and complete formation of the epithelium.	[164]
Polymeric micelles	Curcumin, alginate	Emulsion	In vitro and in vivo with rat model	A concentration of 7.5 mg of curcumin-loaded micelles did not significantly reduce cell viability. The minimum inhibitory concentration values of curcumin were 153, 245, and 319 μg/mL against *S. aureus*, *Pseudomonas aeruginosa*, and *E. coli*. In vivo studies revealed that the curcumin-loaded micelles led to an increase in protein, collagen, and TGFβ1 expression.	[145]
	Curcumin, chitosan, alginate, maltodextrin, pluronic F127, pluronic P123, Tween^®^80	Thin-film hydration	In vivo with Bisphenol A-induced diabetic rat model	The blood glucose level and lipid profile of rats were observed to decrease significantly after treatment with curcumin-loaded micelles. Curcumin-loaded polymeric micelles repaired the pancreatic β cells damaged by “Bisphenol A” and prevented diabetic complications.	[146]

## 7. Conclusions

Wound healing, including acute and chronic wounds, continues to be a major clinical challenge for patients and healthcare providers, which directly impact the healthcare system. Curcumin and its derivatives have garnered significant attention due to their anti-inflammatory, antimicrobial, antioxidant, and wound-healing potential. However, curcumin has several limitations, such as poor solubility, chemical instability, rapid metabolism, and short half-life, which challenges its effective delivery and efficacy. To address these issues, advanced curcumin delivery strategies—including films, hydrogels, nanofibrous scaffolds, and polymeric micelles—have been developed as a wound management platform. These approaches enhance curcumin’s solubility, stability, bioavailability, and therapeutic efficacy. These strategic approaches have demonstrated significant potential for accelerating wound healing, especially when combined with antimicrobial therapies for infected wounds. Nanoencapsulation technology for the development of therapeutics offers further advantages, such as targeted delivery, prolonged retention at the wound site, and minimised off-target effects, resulting in better control over the phases of wound healing. Preclinical studies consistently show that these advanced delivery systems enhance granulation tissue formation, angiogenesis, re-epithelization, collagen deposition, and skin regeneration. Although current research is promising, most studies remain in the preclinical phase, highlighting the need for further human clinical trials to confirm these formulations’ safety and therapeutic efficacy. However, future research should focus on advancing clinical trials, scaling production, and addressing the molecular mechanisms by which curcumin influences chronic inflammation and wound repair to improve patient outcomes and provide more effective treatment options.

## Data Availability

The data will be made available upon request.
